# Determinants of loss to follow-up in HIV care among adults on ART at Adama Hospital Medical College, Ethiopia: a case-control study

**DOI:** 10.1186/s12879-025-11946-8

**Published:** 2025-11-04

**Authors:** Temesgen Oljira, Abebe Megerso, Alem Deksisa, Firaol Regea, Tesfu Zewdu

**Affiliations:** 1 Department of Public Health, Adama Hospital Medical College, Adama, Oromia Ethiopia; 2 Department of Nursing, College of Health Sciences, Ambo University, Waliso Campus, Waliso, Oromia Ethiopia; 3 Department of Nursing, College of Health Sciences, Salale University, Fiche, Oromia Ethiopia

**Keywords:** Determinants, LTFU, Adama Hospital Medical College, ART, Adults

## Abstract

**Background:**

In recent decades, the availability of antiretroviral therapy services has grown significantly, resulting in a reduction in both morbidity and mortality rates among patients undergoing treatment. Loss of follow-up remains a significant public health concern in Ethiopia notwithstanding these advancements. Earlier studies on loss to follow-up (LTFU) mainly used medical records and examined various factors that contribute to patient dropout across different regions. However, this study focused on data collected directly from patients. Therefore, this study aimed to identify the determinants of loss to follow-up among adult patients on ART at Adama Hospital Medical College, Oromia, Ethiopia, 2023.

**Methods:**

An unmatched case-control study was conducted on a sample size of 122 cases and 244 controls. Controls were approached using systematic random sampling method. The ART database was used to identify cases who were LTFU from the services for 90 days or longer from their last appointment date during the 24-month period prior to data collection and selected by simple random sampling. Data were collected by an interviewer-administered structured questionnaire by telephone call from cases and face-to-face interview from controls. The data were entered into Epi Info version 7.2 and analysed using SPSS version 26. Binary logistic regression analysis was conducted. The study findings were reported using adjusted odds ratios with a 95% confidence interval, and significance was determined using a P-value less than 0.05.

**Results:**

Not receiving IPT [AOR = 2.69; 95% CI: (1.86, 12.57)], not receiving CPT [AOR = 5.12; 95% CI: (2.60, 24.81)], TB/HIV co-infection [AOR = 8.42; 95% CI: (6.00, 19.59)], perceived stigma and discrimination [AOR = 2.44; 95% CI: (2.27, 13.18)], and not being a member of a PLHIV association [AOR = 4.71; 95% CI: (3.01, 11.26)] were the determinants of loss to follow-up.

**Conclusion:**

Failure to receive IPT and CPT, perceived stigma and discrimination, TB/HIV co-infection, and not being a member of a PLHIV association were the determinants of loss to follow-up among people living with HIV. Relevant authorities should prioritize addressing these factors to enhance patient retention in care and improve treatment outcomes.

**Clinical trial number:**

Not applicable.

**Supplementary Information:**

The online version contains supplementary material available at 10.1186/s12879-025-11946-8.

## Background

Human immunodeficiency virus (HIV) is a major contributor to sickness and mortality on a global scale [1]. An estimated 39 million people were living with HIV, and 1.3 million new infections and 630,000 deaths from HIV-related causes occurred globally in 2022 [2], and Sub-Saharan Africa contributed 76% of all people living with HIV and 75% of the total HIV/AIDS deaths [3]. In Ethiopia, 718,550 people were living with the virus, with 30,000 new infections [4] and 19,743 deaths yearly [5].

The introduction of antiretroviral treatment has been a groundbreaking advancement, significantly reducing transmission and improving outcomes, including quality of life, access to care, and HIV-related mortality [6]. Universally, 21.7 million individuals were getting ART in 2017. Between 2000 and 2017, new HIV infections diminished by 36%, and HIV-related passings diminished by 38% due to ART accessibility in the world [7].

In Ethiopia, 426,000 individuals are right now taking ART [8]. According to the most recent appraisal, there was a need for ART in 2017 for 551,630 adults and 62,194 children beneath the age of 15 in Ethiopia, and the ART scope for individuals over the age of 15 has come to 75%, but the scope remains low (34%) for children [9].

Globally, loss to follow-up (LTFU) is 24.5%, which exceeds the WHO-recommended target of 15% [10], and in sub-Saharan Africa, it ranges from 30% to 60% [11]. LTFU could be a critical deterrent to the success of ART programs in settings with restricted resources [12]. Antiretroviral treatment discontinuation due to loss to follow-up causes drug resistance, reduces the immunological benefit of treatment, and increases AIDS-related hospitalizations, illness, and death [13–15].

It has been recognized as an obstacle to the achievement of sustainable provision of treatment for the three 95% treatment targets of the Joint United Nations Program on HIV/AIDS (UNAIDS) and affects the performance of the third 95 of the UNAIDS 95-95-95 that aimed at achieving 95% of the virological success of patients on ART [16].

The burden of LTFU among patients with HIV is anticipated to be noteworthy in low- and middle-income nations, posturing a genuine hazard to facilitated endeavors to end the HIV plague by accomplishing near-universal antiretroviral treatment [17].

Although clinical and public health achievements of ART require consistent long-term follow-up [18], retention of patients in long-term treatment programs has received less attention since most treatment providers have limited resources to trace LTFU patients [19]. In general, many studies conducted on LTFU using secondary data showed that sociodemographic factors, clinical factors, social factors, personal factors, and health facility-related factors were the determinants of lost to follow-up among adults who were on ART [20–22]. LTFU remains troublesome for HIV patients in spite of the major effect of ART, with a changing predominance over diverse countries: 36.6% in Cameroon [23], 18.16% among ladies in Uganda [24], 19.24% in Nigeria [25], and 15.17% in Ethiopia [21].

While several studies have examined loss to follow-up among adults on ART, most have relied on secondary data sources, which may lack detailed patient-level insights such as reasons for discontinuation or contextual factors influencing adherence. This study addresses that gap by collecting and analyzing primary data directly from patients, allowing for a more nuanced understanding of the determinants of LTFU [26–28]. Understanding the determinants of LTFU at this institution is crucial for improving patient retention and treatment outcomes in HIV care. The findings of this study will also provide valuable insights for program developers, healthcare providers, and policymakers to develop targeted strategies to avert the problem. Therefore, this study aimed to identify the determinants of loss to follow-up among people living with HIV (PLHIV) at Adama Hospital Medical College, Central Oromia, Ethiopia, 2023.

## Methods

### Study area and period

Adama Hospital Medical College (AHMC) is one of the comprehensive specialized hospitals in the Oromia regional state, located in Adama town, which is 99 km away from Addis Ababa, the capital city of Ethiopia. Adama Hospital Medical College is one of the hospitals in Oromia with a high patient load. It started ART service in March 2005 G.C. and has 20,613 ever-enrolled HIV-positive patients and 15,346 who ever started antiretroviral therapy, of which 7,574 are currently active patients on antiretroviral therapy (ART). From this, 198 are children and 7376 are adults; 6773 of them are on the 1st -line medication, 758 of them are on the 2nd -line regimen, and 43 of them are on the 3rd line regimen. Adama Hospital Medical College works as a referral center for surrounding health facilities and handles patients on the 2nd and the 3rd lines of medication [29]. The study was conducted at the AHMC of ART clinic from March 10 to April 12, 2023.

## Study design

A facility-based unmatched case-control study was conducted.

### Population

#### Source population

The source population was all adult people living with HIV/AIDS who had started ART at Adama Hospital Medical College in Central Oromia, Ethiopia.

#### Study population

##### Cases

People living with HIV (PLHIV) who had ever initiated ART (were on treatment for greater than 6 continuous months) and then discontinued ART for ≥ 3 months during the data collection period at Adama Hospital Medical College, Central Oromia, Ethiopia.

##### Controls

People living with HIV (PLHIV) who had been on ART for greater than 6 continuous months and were still on ART during the data collection period at AHMC, Central Oromia, Ethiopia.

#### Study units

##### Cases

Selected adult patients who were lost to follow-up (LTFU) from treatment prior to the 24-month period and controls: Selected adult patients who remained active on treatment during the data collection period.

### Inclusion and exclusion criteria

#### Inclusion criteria

##### Cases

All people living with HIV (PLHIV) aged 15 years or older who had been on ART for more than 6 uninterrupted months and then discontinued ART for ≥ 3 months.

##### Controls

All people living with HIV (PLHIV) aged 15 years or older who have been on ART for greater than 6 continuous months and are currently active on ART at Adama Hospital Medical College, Central Oromia, Ethiopia.

#### Exclusion

##### Cases

Individuals who discontinued ART for ≥ 3 months and did not have a registered telephone number at AHMC were excluded.

##### Controls

Individuals with a history of discontinuation who are currently active on treatment were excluded.

#### Sample size determination

Sample size was calculated using Epi Info version 7.2 software with the following assumptions: a 95% confidence level, 80% power, a two-to-one ratio of controls to cases (2:1), and by taking statistically significant determinants of LTFU from a previous study conducted in the Oromia region [26]. Suboptimal (F/P) adherence was chosen as the primary exposure variable, as it yielded the maximum sample size compared to other exposure variables. Consequently, the sample size for cases and controls was 111 and 222, respectively. After accounting for a 10% non-response rate, the final sample size was adjusted to 122 cases and 244 controls.

#### Sampling procedures

The ART database was used to extract the information for all adult patients on ART who had LTFU appointments for 3 months or longer from their last appointment date during the 24-month period prior to the data collection. All eligible controls were selected through systematic random sampling from a total of seven thousand three hundred seventy-six individuals, with a calculated k value of thirty, while cases were selected via simple random sampling from one hundred sixty-two individuals. Their unique ART number was used as the sampling frame for cases and controls.

### Data collection tools and procedure

A structured questionnaire was initially prepared in the English language after reviewing relevant published literature [6, 26, 28, 30, 31] (Supplementary file). The questionnaire was then translated into Afan Oromo and Amharic language for data collection purposes and later translated back into English to ensure consistency. Eligible individuals, both cases and controls, were identified, and consent was obtained from them. Data collection for cases was conducted via phone calls, while controls were interviewed face-to-face. The data were collected by four BSc nurse professionals who had received comprehensive ART care training and had prior experience working in an ART clinic. Additionally, a diploma holder in information technology assisted in retrieving patients’ unique ART numbers from the electronic medical records. Two BSc nurses interviewed the controls, and the other two conducted phone interviews with the cases. The data collection process was closely supervised by a Master of Public Health professional. To ensure the standardization of responses and avoid bias between face-to-face and phone interviews during data collection, the following strategies were implemented: Training of Interviewers: All interviewers received standardized training on the interview protocol. This included clear guidelines on how to ask questions, handle sensitive topics, and address any misunderstandings or clarifications. This ensured that the same questions were asked in a consistent manner and reduced potential bias from interviewers. Pretest: Before starting the actual data collection, a pretest was conducted for both face-to-face and phone interviews to identify any potential differences in how questions were interpreted or answered. An adjustment was made based on the results of the pretest. Monitoring and supervision: The supervisor strictly monitored both face-to-face and phone interviews to ensure adherence to the interview guidelines. Post-interview data verification: After the completion of phone interviews, the supervisor reviewed a sample of the collected data to verify accuracy. Cross-checking: Some interviews were cross-checked by contacting participants via phone after a face-to-face interview or vice versa.

### Study variables

#### Dependent variable

Loss to Follow Up from ART.

### Independent variables

**Socio-demographic factors** (sex, age, educational status, marital status, occupation, place of residency, having an HIV-positive family member, and household income).

**Clinical factors** (functional status, baseline BMI, baseline CD4 count, opportunistic infection (OI), isoniazid prophylaxis therapy (IPT), cotrimoxazole preventive therapy (CPT), and baseline WHO clinical stage).

**Behavioral/personal factors** (status disclosure, use of traditional medicine).

**Health services factors** (waiting time at clinics during visits, availability of health workers, convenience of appointment schedule, and pre-ART counselling).

**Social factors** (stigma, experiences of bereavement, and membership in a People Living with HIV (PLHIV) association).

### Operational definitions

Loss to follow-up: The patient fails to visit ART clinics for ≥ 3 months after the last appointment date and was not classified as either “died” or “transferred.

Transfer out: when a patient is referred from the facility where s/he started ART to another health facility.

Detected viral load: Viral load above 1000 copies/mL.

Not detected: viral load less than or equal to 1000 copies/mL.

Baseline clinical conditions are defined as the value closest to the ART start date, not exceeding 3 months prior to the start date.

Functional status: The patient status of “working” if daily activities of PLHIV were not altered due to illness, “ambulatory” if the patient was not fully working but was able to do minor tasks at home, and “bedridden” when the patient remained in bed most of the time.

Disclosure – Occurs when the status of the individual is disclosed to at least one individual.

ART adherence – Defined as the percentage of ART drug dosage taken from a monthly dose was classified as good, fair, or poor. Hence, good adherence was reported if equal to or greater than 95% (≤ 3 doses missing per month), fair if 85–94% (4–9 doses missing per month), or poor if less than 85% (> 9 doses missing per month).

Adults: In the ART department, adults are defined as people living with HIV (PLHIV) who are 15 years of age or older.

Traditional medicines: Locally available substances provided by non-health professionals, which are taken by people living with HIV orally, through inhalation, and applied to the body for medicinal purposes.

Social support: An integral component of the holistic approach to care, provided by individuals or communities to support the well-being and care of people living with HIV (PLHIV).

### Data quality assurance

A two-day training was given for data collectors and supervisor on the objectives and process of data collection prior to the start of the study. Pretest was done on 5% of the same source population, which was not included in the final sample size. The collected data were checked for consistency and completeness on a daily basis by the supervisor. Data were double-entered into Epi-info version 7.2 to ensure data quality.

### Data analysis

The collected data were coded and entered into Epi Info version 7.2 and then exported to SPSS version 26 for analysis. A binary logistic regression model was used to identify the determinants of LTFU. Bivariate and multivariate logistic regression analyses have been performed. Tables and text were used to present the results of the analysed data. Independent variables with a P-value of < 0.25 in bivariate logistic regression analysis were considered for inclusion in the multivariate logistic regression analysis, and a P-value < 0.05 was used as a cutoff to declare the statistical significance. Multicollinearity was assessed using the variance inflation factor, which was found to be less than 10. Finally, model goodness of fit was checked using the Hosmer Lemeshow test of goodness of fit (P-value > 0.05).

## Results

### Socio-demographic related factors of study participants

From a total of 366 (122 cases and 244 controls), 359 (120 cases and 239 controls) were included in the study, resulting in a 98% response rate for both cases and controls. Out of the total participants, 71 (59.2%) of the cases and 143 (59.8%) of the controls were male. The median age for cases was 40 years (IQR: 30–45), while the median age for controls was 43 years (IQR: 37–50). Forty-five (37.5%) of the cases and 92 (38.5%) of the controls belonged to the age category of 35 to 44. Ninety-eight (81.7%) of the cases and 198 (82.8%) of the controls were urban residents. Regarding marital status, 44 (36.7%) of the cases and 146 (61.1%) of the controls were married (Table 1).

**Table 1:** Socio-demographic characteristics of people living with HIV at AHMC, Oromia, Ethiopia, 2023 (*n* = 359)

Variables	Respondent status			
	Cases(*n* = 120)		Controls(*n* = 239)	
	Number	%	Number	%
**Age**				
15 to 34	43	35.8	40	16.7
35 to 44	45	37.5	92	38.5
45 and above	32	26.7	107	44.8
**Sex**				
Male	71	59.2	143	59.8
Female	49	40.8	96	40.2
**Educational status**				
No formal education	40	33.3	49	20.5
Primary	35	29.2	79	33.1
Secondary and above	45	37.5	111	46.4
**Marital status**				
Married	44	36.7	146	61.1
Single	76	63.3	93	38.9
**Residence**				
Urban	98	81.7	198	82.8
Rural	22	18.3	41	17.2
**Religion**				
Orthodox	76	63.3	162	67.8
Protestant	23	19.2	45	18.8
Muslim	21	17.5	32	13.4
**HIV positive family members**				
No	70	58.3	163	68.2
Yes	50	41.7	76	31.8
**Household income per month**				
1000 to 5000	75	62.5	145	60.7
5001 and above	45	37.5	94	39.3

### Clinical related factors

The majority of participants, 114 (95%) of the cases and 225 (94.1%) of the controls, had been on ART for ≥ 12 months. The median baseline body mass index for cases was 18.3 kg/m² (IQR: 16.4–19.8), while for the controls, it was 18.9 kg/m² (IQR: 17.4–21.5). Fifty-five (45.8%) of the cases and 105 (43.9%) of the controls had a body mass index less than 18.5. Regarding baseline WHO clinical stage, 50 (41.7%) of the cases and 92 (38.5%) of the controls were in WHO clinical stage III. The median baseline CD4 cell count of the cases was 212.5 cells/mm3 [IQR: 102.25-331.75 cells/mm3], and for the controls, it was 220 cells/mm3 [IQR: 119–349 cells/mm3]. Nearly one-fourth, 26 (21.7%) of the cases, and 40 (16.7%) of the controls had a baseline CD4 count > 350 cells/mm³. Regarding IPT status, 83 (69.2%) of the cases and 24 (10.04%) of the controls had no history of receiving IPT prophylaxis. Thirty-one (25.8%) of the cases and 162 (67.8%) of the controls had a history of receiving cotrimoxazole. Nearly all cases, 107 (89.2%) of the cases, and 93 (38.9%) of the controls had a history of TB/HIV co-infection (Table 2).

**Table 2:** Clinical characteristics of people living with HIV at AHMC, Oromia, Ethiopia, 2023 (*n* = 359)

Variables	Respondent status			
	Cases(*n* = 120)		Controls(*n* = 239)	
	Number	%	Number	%
**BaselineCD4**				
< 200	55	45.8	103	43.1
200 to 350	39	32.5	96	40.2
> 350	26	21.7	40	16.7
**BMI kg/m2**				
< 18.5	55	45.8	105	43.9
>=18.5	65	54.2	134	56.1
**Baseline WHO stage**				
Stage one	31	25.8	75	31.4
Stage two	31	25.8	59	24.7
Stage three	50	41.7	92	38.5
Stage four	8	6.7	13	5.4
**IPT status**				
Don’t use IPT	83	69.2	24	10.04
Use IPT	37	30.8	215	89.96
**Recent viral load**				
> 1000copies/mm3	84	70	4	1.7
<=1000copies/mm3	14	11.7	232	97.1
Don’t done	22	18.3	3	1.2
**Received CPT**				
No	89	74.2	77	32.2
Yes	31	25.8	162	67.8
**History of TB/HIV co-infection**				
No	13	10.8	146	61.1
Yes	107	89.2	93	38.9

### Health services related factors

The median waiting time for the cases was 45 min [IQR: 30–60], while for the controls, it was 60 min (IQR: 35–60). Fifty-eight (48.3%) of the cases and 144 (60.3%) of the controls reported a waiting time of 31–60 min.

The majority, 90 (75%) of the cases and 203 (84.9%) of the controls, had received pre-ART counselling. Additionally, 110 (91.7%) of the cases and 226 (94.6) of the controls reported never facing unavailability of healthcare workers during clinic visits. Furthermore,113 (94.2%) of the cases and 218 (91.2%) of the controls had convenient appointment schedules or times (Table 3).

**Table 3:** Health services related factors of people living with HIV at AHMC, Oromia, Ethiopia, 2023 (*n* = 359)

Variables	Respondent status			
	Cases(*n* = 120)		Controls(*n* = 239)	
	Number	%	Number	%
**Waiting time in minutes at the** **clinic**				
15 to 30	33	27.5	60	25.1
31 to 60	58	48.3	144	60.3
61 to 120	29	24.2	35	14.6
**Have you ever got pre-ART counselling?**				
No	30	25	36	15.1
Yes	90	75	203	84.9
**Have you ever faced unavailability of health workers during visit?**				
No	110	91.7	226	94.6
Yes	10	8.3	13	5.4
**Is the appointment time being convenient**				
No	7	5.8	21	8.8
Yes	113	94.2	218	91.2

### Personal related factors

Seventy-four (61.7%) of the cases and 232 (97.1%) of the controls disclosed their HIV status to their partners/parents. Among those who disclosed their status, nearly half, 59 (79.7%) of the cases and 184 (79.3%) of the controls, disclosed it to their partners. Additionally, 68 (56.7%) of the cases and 10 (4.2%) of the controls had ever used traditional medicine (Table 4).

**Table 4:** Personal characteristics of people living with HIV at AHMC, Oromia, Ethiopia, 2023

Variables	Respondent status			
	Cases(*n* = 120)		Controls(*n* = 239)	
	Number	%	Number	%
Disclosure status				
No	46	38.3	7	2.9
Yes	74	61.7	232	97.1
If yes for the above question for whom the participant discloses				
Wife/husband	59	79.7	184	79.3
Family members and others	15	20.3	48	20.7
Have you ever used traditional medicine				
No	52	43.3	229	95.8
Yes	68	56.7	10	4.2

**Social related factors**

Ninety-one (75.8%) of the cases and 74 (31.0%) of the controls were not members of a people living with HIV (PLHIV) association. Fifty-five (45.8%) of the cases and 115 (48.1%) of the controls reported having social support.

Twenty (16.7%) of the cases and 47 (19.7%) of the controls had faced bereavement. Additionally, 51 (42.5%) of the cases and 63 (26.4%) of the controls reported experiencing perceived stigma and discrimination (Table 5).

**Table 5:** Social factors of people living with HIV at AHMC, Oromia, Ethiopia, 2023 (*n* = 359)

Variables	Respondent status			
	Cases(*n* = 120)		Controls(*n* = 239)	
	Number	%	Number	%
Are you a member of the Association of people living with HIV				
No	91	75.8	74	31.0
Yes	29	24.2	165	69.0
Have you ever received/got social support				
No	65	54.4	124	51.9
Yes	55	45.8	115	48.1
Have you faced Bereavement due to HIV				
No	100	83.3	192	80.3
Yes	20	16.7	47	19.7
Have you ever perceived stigma and discrimination while you have been taking ART				
No	69	57.5	176	73.6
Yes	51	42.5	63	26.4

### Determinants of loss to follow-up in HIV care among adults on ART at Adama Hospital Medical College, Central Oromia, Ethiopia

In the bivariate logistic regression analysis, factors such as marital status, having a family history of HIV-positive, not receiving IPT, not receiving CPT, history of TB/HIV co-infection, receiving pre-ART counselling, perceived stigma and discrimination, and not being a member of people living with HIV/AIDS were found to be the determinants of LTFU at a P-value less than 0.25.

In multivariate logistic regression analysis, factors such as not receiving IPT, not receiving CPT, history of TB/HIV co-infection, perceived stigma and discrimination, and not being a member of PLHIV were statistically the determinants of lost to follow-up at a P-value less than 0.05 (Table 6). The odds of not receiving IPT among cases was 2.69 times higher compared to controls [AOR = 2.69; 95% CI: (1.86, 12.57)]. The odds of not receiving CPT among cases was 5.12 times higher compared to controls [AOR = 5.12; 95% CI: (2.60, 24.81)]. The odds of having a history of TB/HIV co-infection among cases was 8.42 times higher compared to controls [AOR = 8.42; 95% CI: (6.00, 19.59)]. Additionally, the odds of perceiving HIV stigma and discrimination among cases was 2.44 times higher compared to controls [AOR = 2.44; 95% CI: (2.27, 13.18)], and lastly, the odds of not being a member of a PLHIV association among cases was 4.71 times higher compared to controls [AOR = 4.71; 95% CI: (3.01, 11.26)].

**Table 6:** Determinants of loss to Follow-Up in HIV care among adults on ART at Adama hospital medical College, 2023

Determinants	Lost to Follow Up		COR 95% CI	AOR 95% CI
	Cases (%)	Controls (%)		
**Marital status**				
Married	44(36.7)	146(61.1)	0.37(0.12, 4.267)	0.25(0.101,2.00)
Single	76(63.3)	93(38.9)	1	
**HIV positive family member**				
No	70(58.3)	163(68.2)	0.65(0.35, 5.4)	1.34(0.557,3.227)
Yes	50(41.7)	76(31.8)	1	
**Received IPT**				
Don’t use IPT	83(69.2)	24(10.04)	20.09(11.33,35.63)	2.69(1.86,12.57) ***
Use IPT	37(30.8)	215(89.96)	1	
**Received CPT**				
No	89(74.2)	77(32.2)	6.04(1.035, 17.29)	5.12(2.60,24.81) ***
Yes	31(25.8)	162(67.8)	1	
**Current Viral load**				
Not done	22(18.3)	4(1.7)	0.196(0.02,14.95)	2.401(0.812,3.242) 0.04(0.02,4.143)
<=1000	14(11.7)	232(97.1)	0.002(0.001,21.6)	
>1000	84(70.0)	3(1.2)	1	
**TB/HIV Co-infection**				
Yes	107(89.2)	93(38.9)	12.92(8.21, 41.73)	8.42(6.00,19.59) ***
No	13(10.8)	146(61.1)	1	
**Received pre-ART counselling**				
No	30(25)	36(15.1)	1.88(1.36,20.24)	0.308(0.087,5.062)
Yes	90(75)	203(84.9)	1	
**Perceived stigma and discrimination**				
Yes	51(42.5)	63(26.4)	2.06(1.80, 6.002)	2.44(2.27, 13.18) ***
No	69(57.5)	176(73.6)	1	
**Member of PLHIV**				
No	91(75.8)	74(31.0)	6.99(2.069, 19.189)	4.71(3.01, 11.26) ***
Yes	29(24.2)	165(69.0)	1	

Note: ***represents *P* < 0.05

## Discussion

The objective of this study was to identify the determinants of lost to follow-up in HIV care among adults on ART at Adama Hospital Medical College. To begin with, it was found that the odds of not receiving IPT among cases was 2.69 times higher compared to controls. This is consistent with studies conducted in Nigeria [32], India [19], and Oromia, Gondar Hospital, and Pawi General Hospital [26, 33, 34]. This could be due to the fact that the start of IPT, which is suggested by the national ART guideline, might straightforwardly diminish LTFU by expanding the maintenance of HIV patients on care since IPT prevents the event of tuberculosis co-infection, which is the foremost life-threatening opportunistic infection among individuals living with HIV [35].

The odds of not receiving CPT among cases was 5.12 times higher compared to controls. This finding was comparable with studies conducted in Gondar, Ethiopia, and Nigeria [32, 33].

The possible explanation for this result might be due to the effect of CPT on reducing the incidence of many opportunistic infections such as pneumocystis pneumonia, toxoplasmosis, bacterial infections, & diarrheal diseases, which may indirectly improve the lived experience of the patient on ART, and this suggests that preventing the occurrence of opportunistic infections through widespread access to prophylaxis therapy enhances patient’s retention in HIV care [36].

It was found that the odds of having a history of TB/HIV co-infection among cases was 8.42 times higher compared to controls. This result is consistent with findings from earlier studies in Cameron and Ethiopia at Felegehiwot Hospital [37, 38]. This is because TB increases HIV replication through the process of immune activation, leading to an increased viral load. This results in a more rapid progression of HIV disease, which might make people bedridden. TB enables the progression of HIV disease to an advanced stage quickly, thereby barring patient adherence. In addition, TB/HIV co-infection could be another factor for ART interruption due to pill burden [9]. However, this finding is different from the study conducted in Botswana [39]. The difference with our study is that we looked at proven cases of TB, confirmed by microscopy, whereas the Botswana study used the past history of both presumed and confirmed TB cases.

Additionally, the odds of perceiving HIV stigma and discrimination among cases was 2.44 times higher compared to controls. This was supported by studies conducted in Namibia, Zambia, Oromia, and Tigray region [18, 28, 30, 37]. However, this finding is different from the study conducted in Botswana [39]. The differences in findings between the two studies may be due to the types of data used in the analysis. The previous study relied on secondary data, while this study used primary data. Secondary data may introduce biases such as incomplete records, data reporting issues, or a lack of control over the data collection process.

The odds of not being a member of the PLHIV association among cases was 4.71 times higher compared to controls. This was in line with a study conducted in East Gojjam Zone [31]. A possible explanation is that being a member of a People Living with HIV (PLHIV) association may provide individuals with the opportunity to share their experiences and take a more active role in managing their care. The limitations of this study include recall bias or the use of different methods for data collection (phone calls for cases and face-to-face interviews for controls), which may lead to slight variations in responses that might influence the results.

## Conclusion and recommendation

In this study, failure to receive IPT and CPT, perceived stigma and discrimination, TB/HIV co-infection, and not being a member of a PLHIV association were the main determinants of LTFU among people living with HIV. Therefore, the regional health bureau, town health office, and Adama Hospital Medical College should strengthen community-based adherence counselling to improve IPT/CPT uptake, expand peer-led PLHIV support groups to encourage retention, and implement targeted interventions for patients with TB/HIV co-infection.

## Supplementary Information

Below is the link to the electronic supplementary material.

Supplementary Material 1

## Data Availability

The data used in this study are available from the corresponding author on rational request.

## Abbreviations

AIDS: Acquired Immune-Deficiency Syndrome
AHMC: Adama Hospital Medical College
AOR: Adjusted Odds Ratio
ART: Antiretroviral Therapy
ARV: Anti-Retroviral
BMI: Body Mass Index
CD4: Cluster Differentiation 4
CI: Confidence Interval
CPT: Cotrimoxazole Preventive Therapy
LTFU: Loss to Follow Up, OIS: Opportunistic Infection
PLWHIV: People Living With HIV/AIDS
SPSS: Statistical Packages for social science
TB: Tuberculosis
UNAIDS: United Nation’s Program on HIV/AIDS
WHO: World Health Organization
