# Supplementary material for: Determinants of loss to follow-up in HIV care among adults on ART at Adama Hospital Medical College, Ethiopia: a case-control study
Source: BMC Infect Dis. 2025 Nov 4;25:1489. doi: 10.1186/s12879-025-11946-8 (PMC12584480; doi:10.1186/s12879-025-11946-8)
Supplement: Supplementary file 1 — Supplementary Material 1 [file 12879_2025_11946_MOESM1_ESM.docx]

**Questionnaire**

**Section One**: Sociodemographic characteristics

| S. No | Question | Responses | Remark |
| --- | --- | --- | --- |
| 101 | Age | ………. years (in complete years) |  |
| 102 | Sex | 1. Female 2. Male |  |
| 103 | Educational status | 1. No formal education 2. Primary 3. Secondary 4. Certificate and above |  |
| 104 | Marital status | 1. Married 2. Single 3. Divorced 4. Separated 5. Widowed |  |
| 105 | Residence | 1. Urban 2. Rural |  |
| 106 | Religion | 1. Orthodox 2. Catholic 3. Muslim 4. Protestant 5. Others, specify... |  |
| 107 | Occupation | 1. Farmer 2. Student 3. House wife 4. Merchant 5. Governmental/nongov’tal employee 6. Daily labourer 7. Other, specify…... |  |
| 108 | HIV positive family members | 1. Yes 2. No |  |
| 109 | House hold income | _______Birr |  |

**Section Two**: Clinical related characteristics

| 201 | | Baseline CD4 (cell/µl) | ---------- | Remark | |  |
| --- | --- | --- | --- | --- | --- | --- |
| 202 | | Baseline BMI in kg/m2 | ---------- |  | |  |
| 203 | | Baseline WHO stage | 1. Stage one 2. Stage two 3. Stage three 4. Stage four |  | |  |
| 204 | | Months on ART | 1. <12 Month 2. ≥12 Month |  | |  |
| 205 | | IPT status | 1. Don’t use IPT 2. Use IPT |  | |  |
| 206 | | Received CPT | 1. Yes 2. No |  | |  |
| 207 | | Viral Load count | 1. >1000 copies/mm3 2. =<1000 copies/mm3 3. Not done |  | |  |
| 208 | | History of TB/ HIV co-infection | 1. Yes 2. No |  | |  |
| 209 |  | History of opportunistic infections  other than TB | 1. Yes 2. No | |  | |
| 210 |  | Baseline functional status | 1. Working 2. Bedridden 3. Ambulatory | |  | |
| 211 |  | What was the adherence status of HAART? | 1. Good 2. Fair 3. Poor | |  | |
| 212 |  | The outcome of the patient | 1. LTFU 2. On follow up 3. Other, specify……... | |  | |
|  | | | | | |  |

**Section Three**: Health Services related Factors

| 301 | | Have you ever got pre- ART counselling? | 1. Yes  2. No | If the answer is no, skip to Q303 |
| --- | --- | --- | --- | --- |
| 302 | | If Q301 is yes, what was your satisfaction? | 1. More satisfied  2. Moderately satisfied  3. Satisfied  4. Not satisfied |  |
| 303 | | Waiting time (in munities) of the recent visit at clinics | ------------ |  |
| 304 | | Have you ever faced unavailability of health providers during visits? | 1. Yes  2. No |  |
| 305 | Is the appointment time’s convenience? | | 1. Yes  2. No |  |
| 306 | Have you been satisfied with services received at the facility? | | 1. More satisfied  2. Moderately satisfied  3. Satisfied  4. Not satisfied |  |

**Section Four**: Personal related factors

| 401 | Disclosure status | 1. Yes 2. No | If No, skip to Q403 |
| --- | --- | --- | --- |
| 402 | If yes for question No.401, for  whom the participant discloses | 1. Wife/husband 2. Family members 3. Neighbours 4. Friends 5. Others, specify…. |  |
| 403 | Have you ever used traditional medicine? | 1. Yes  2. No |  |

**Section Five**: Social related Factors

| S. No. | Question | Response |  |
| --- | --- | --- | --- |
| 501 | Have you ever perceived stigma and discrimination while you have been taking ART drugs? | 1. Yes  2. No |  |
| 502 | Are you a member of the Association of PLHIV? | 1. Yes  2. No |  |
| 503 | Have you ever got social support? | 1. Yes  2. No |  |
| 504 | Have you faced Bereavement due to HIV? | 1. Yes  2. No |  |
